# Risk Factors for Death from Visceral Leishmaniasis in an Urban Area of Brazil

**DOI:** 10.1371/journal.pntd.0003982

**Published:** 2015-08-14

**Authors:** Angelita F. Druzian, Albert S. de Souza, Diogo N. de Campos, Julio Croda, Minoru G. Higa, Maria Elizabeth C. Dorval, Mauricio A. Pompilio, Polliana A. de Oliveira, Anamaria M. M. Paniago

**Affiliations:** 1 University Hospital, Federal University of Mato Grosso do Sul, Campo Grande, Mato Grosso do Sul, Brazil; 2 Post Graduation Program in Infectious and Parasitic Diseases, Federal University of Mato Grosso do Sul, Campo Grande, Mato Grosso do Sul, Brazil; 3 Center for Biological and Health Sciences, Federal University of Mato Grosso do Sul, Campo Grande, Mato Grosso do Sul, Brazil; 4 Medicine Faculty, Federal University of Mato Grosso do Sul, Campo Grande, Mato Grosso do Sul, Brazil; 5 Oswaldo Cruz Foundation—Fiocruz, Mato Grosso do Sul, Campo Grande, Mato Grosso do Sul, Brazil; 6 Faculty of Health Sciences, Federal University of Grande Dourados, Dourados, Mato Grosso do Sul, Brazil; Hospital Universitário Professor Edgard Santos, BRAZIL

## Abstract

**Background:**

Over the last three decades, the epidemiological profile of visceral leishmaniasis (VL) has changed with epidemics occurring in large urban centers of Brazil, an increase in HIV/AIDS co-infection, and a significant increase in mortality. The objective of this study was to identify the risk factors associated with death among adult patients with VL from an urban endemic area of Brazil.

**Methodology:**

A prospective cohort study included 134 adult patients with VL admitted to the University Hospital of the Federal University of Mato Grosso do Sul between August 2011 and August 2013.

**Principal Findings:**

Patients ranged from 18 to 93 years old, with a mean age of 43.6 (±15.7%). Of these patients, 36.6% were co-infected with HIV/AIDS, and the mortality rate was 21.6%. In a multivariate analysis, the risk factors associated with death were secondary bacterial infection (42.86, 5.05–363.85), relapse (12.17, 2.06–71.99), edema (7.74, 1.33–45.05) and HIV/AIDS co-infection (7.33, 1.22–43.98).

**Conclusions/Significance:**

VL has a high mortality rate in adults from endemic urban areas, especially when coinciding with high rates of HIV/AIDS co-infection.

## Author Summary

Visceral Leishmaniasis (VL) is considered a neglected disease by the World Health Organization (WHO). Over the last two decades, the epidemiological profile of VL has changed with epidemics occurring in large urban centers of Brazil with increased HIV/AIDS co-infection and mortality. Understanding the factors that lead to death in these patients is important to improve public health strategies and reduce mortality. We performed a prospective cohort study between August 2011 and August 2013 of 134 adult patients with VL from Campo Grande urban area. Approximately 40% had AIDS and 22% died during the 12-month follow-up. Relapses, bacterial infection, AIDS and edema were strongly associated with death among VL patients in the Brazilian urban centers. All VL patients should be screened for HIV and bacterial infection as well as to prevent VL relapses.

## Introduction

Over 90% of visceral leishmaniasis (VL) cases occur in six countries: Bangladesh, Brazil, Ethiopia, India, South Sudan and Sudan [1]. In Brazil, an increase in incidence over the past three decades has coincided with epidemics in large urban centers such as Campo Grande, Teresina, São Luis, Natal and Belo Horizonte [2] and a significant increase in mortality [3].

Despite increased mortality, there are few studies assessing risk factors associated with death among VL patients and the majority involves populations of all age groups in urban and non-urban areas with low rates of HIV co-infection. In non HIV infected patients, jaundice, bleeding, and associated infections were most frequent risk factors [4–8], while being older than 45 years of age was reported by one study [9].

In addition, over the last three decades, Brazil has had an increase in the HIV/AIDS epidemic [10]. Due to the changes in the epidemiological profile of VL and its expansion into urban areas with higher prevalence of HIV/AIDS, prospective studies are necessary to better understand the determinants associated with death and to propose future interventions. This study aims to identify risk factors associated with death from VL in adult patients from endemic urban areas of Brazil.

## Materials and Methods

### Type of study, location and population

This prospective cohort study was conducted between August 2011 and August 2013 with adult patients with VL. Patients were monitored by the Reference Service for Infectious and Parasitic Diseases of the University Hospital in the Federal University of Mato Grosso do Sul, Campo Grande, Mato Grosso do Sul, Brazil. Clinical, sociodemographic and laboratory variables were recorded on a standardized form and. The criteria for inclusion in the study were for patients to be aged 18 years or older and have a confirmed laboratory diagnosis of VL.

### Diagnostic procedures

The VL diagnosis was performed according to the recommendations of the laboratory of the Ministry of Health [11]. Parasitological diagnoses were performed based on detection of amastigotes of the parasite in Giemsa stained bone marrow smears by an experienced microscopist and by isolation of promastigotes from culture media (Mc Neal, Novy & Nicolle-NNN and Brain Heart Infusion-BHI). Immunological tests were performed using an immunochromatographic test (Kala-azar Detect, INBIOS, WA). Cases were considered confirmed if they presented at least one positive laboratory test, parasitological or immunochromatographic test. HIV screening was performed by enzyme-linked immunosorbent assay and confirmed with an immunofluorescence antibody test, or Western Blot assay [11].

### Protocol of treatment

The treatment protocol was defined according to the guidelines of the Ministry of Health and considered severity signs (jaundice, bleeding hemorrhages, generalized edema, signs of toxemia and severe malnutrition), co-infection with HIV/AIDS, age, renal failure and heart disease [12, 13].

Patients with less than 50 years, without HIV infection, without renal failure or heart disease and without severity signs were treated with antimoniate of N-methylglucamine, which contains pentavalent antimony (Sb^v^), at a dose of 20 mg/kg/day of Sb^v^ for 30 days. Patients with severity signs received amphotericin B deoxycholate at a dose of 1 mg/kg/day for 14 to 20 days [14].

Patients with more than 50 years or with HIV/AIDS or renal or cardiac failure, with or without severity sign, received liposomal amphotericin B at 4 mg/kg/day for 5 days or 3 mg/kg/day for 7 days with 20 mg/kg of total dose [13].

### Follow-up

After discharge, patients were instructed to return after 1, 3, 6 and 12 months for clinical and laboratory revaluation.

### Protocol of secondary prophylaxis

After treatment, patients with AIDS received secondary prophylaxis of liposomal amphotericin B, administered at 3 mg/Kg every 15 days to avoid relapse [12, 15].

### Relapse definition

Relapse was defined as showing clinical signs and symptoms suggestive of VL within 12 months in successfully treated patients [11]. Successful therapy was achieved if there was improvement of general condition, resolution of fever, regression of splenomegaly and recovery of blood counts [15].

### Statistical analysis

Data were tabulated in a spreadsheet (S1 Data). SAS version 9.2 (SAS Institute, Cary, NC) and SPSS version 22.2 were used to analyze bivariate and multivariable models. Dichotomy or categorical data were analyzed with the chi-square test or Fisher’s exact test. For continuous variables, the t-test or Anova was utilized. Missing data were excluded of analyses. Univariate and multivariate logistic regressions were conducted to identify factors associated with death. Variables were included in the model if they achieved a significance level of *p*<0.20 in the univariate analysis. Correlated variables were tested individually, and the Wald test was used to evaluate the significance level of risk factors in the final model. The results were expressed as relative risk (RR) with 95% confidence intervals (95% CI). A correlation matrix was used to assess confounding variables and correlations between variables. Kaplan-Meier survival curves were constructed for each variable using the long-rank test. Statistical significance was set at *p*<0.05.

### Ethical considerations

The present study was approved by the Ethics Committee of the Federal University of Mato Grosso do Sul under protocol number 2179 and case number 02480049000–11. All patients voluntarily signed a statement of informed consent for the collection of data.

## Results

One hundred thirty-seven patients were eligible for the study, however, three not agreed to participate. Of the 134 participants in the present study, 94 (70.1%) were male. The age of participants ranged from 18 to 93 years, with a mean age of 43.6 (SD 41 ± 15.7). Most of the individuals (95%) were from urban areas, 107 (79.8%) from Campo Grande, and the remainder from 17 other cities.

The most common comorbidity in VL patients was AIDS (49;36.6%), followed by tuberculosis (3;2.3%), erythematosus systemic lupus (1;0.75%) and chronic myelocytic leukemia (1;0.75%). At the time of diagnosis of VL in patients with HIV/AIDS, the mean T-CD4+ cell count of was 68/mm^3^. VL and HIV/AIDS were diagnosed simultaneously in 32.7% of the patients.

Parasitological examination (direct and culture) was performed in 126 (94.0%) patients, with 93 positive results (73.84%). Of the 119 (88.8%) samples tested by immunochromatographic test, 96 (80.7%) were positive. In 57 (42.5%) patients, diagnosis was confirmed by two methods (parasitological and immunochromatographic test), only by parasitological in 36 (26.9%), and only by immunochromatographic test in 41 (30.6%) patients.

The time between the first symptoms and diagnosis of VL ranged from one to 421 days, with a median of 31 days. The period between diagnosis and death ranged from three to 431 days, with a median of 346 days. The mortality rate was 21.6% (29/134). Differences in demographic, clinical, laboratory and therapeutic relation to the evolution of the disease are shown in Tables 1 and 2.

**Table 1:** Adult patients with visceral leishmaniasis according to the following aspects: demographic; clinical; therapeutic; co-morbidities and evolution to death (n = 134).

Variable		Death		RR	CI 95%	*P* value
	Total	Yes	No			
	(n = 134)	(n = 29)	(n = 105)			
**Gender**						
**Male**	70.1 (94)	18.1 (17)	81.9 (77)	1		0.192
**Female**	29.9 (40)	30.0 (12)	70.0 (28)	1.66	0.88–3.15	
**Age in years**						
**18 to 50**	74.6 (100)	19.0 (19)	81.0 (81)	1		0.302
**≥ 50**	25.4 (34)	29.4 (10)	70.6 (24)	1.55	0.80–2.99	
**Fever**						
**No**	9.1 (12)	50.0 (6)	50.0 (6)	1		**0.029**
**Yes**	90.9 (120)	18.3 (22)	81.7 (98)	0.37	0.19–0.72	
**Edema**						
**No**	65.9 (87)	11.5 (10)	88.5 (77)	1		**<0.001**
**Yes**	34.1 (45)	37.8 (17)	62.2 (28)	3.29	1.64–6.57	
**Splenomegaly**						
**No**	34.6 (46)	39.1 (18)	60.9 (28)	1		**<0.001**
**Yes**	65.4 (87)	11.5 (10)	88.5 (77)	0.29	0.15–0.58	
**Hepatomegaly**						
**No**	29.1 (39)	35.9 (14)	64.1 (25)	1		**0.019**
**Yes**	70.9 (95)	15.8 (15)	84.2 (80)	0.44	0.24–0.82	
**Cough**						
**No**	37.6 (50)	16.0 (8)	84.0 (42)	1		0.298
**Yes**	62.4 (83)	25.3 (21)	74.7 (62)	1.58	0.76–3.30	
**Weight loss**						
**No**	9.2 (12)	0.0 (0)	100.0 (12)	1		0.127
**Yes**	90.8 (119)	23.5 (28)	76.5 (91)	-	-	
**Relapse**						
**No**	82.8 (111)	15.3 (17)	84.7 (94)	1		**<0.001**
**Yes**	17.2 (23)	52.2 (12)	47.8 (11)	3.41	1.89–6.13	
**HIV**						
**No**	63.4 (85)	11.8 (10)	88.2 (75)	1		**<0.001**
**Yes**	36.6 (49)	38.8 (19)	61.2 (30)	3.30	1.67–6.51	
**Bacterial infection**						
**No**	64.9 (87)	6.9 (6)	93.1 (81)	1		**<0.001**
**Yes**	35.1 (47)	48.9 (23)	51.1 (24)	7.10	3.11–16.20	
**Pneumonia**						
**No**	87.3 (117)	15.4 (18)	84.6 (99)	1		**<0.001**
**Yes**	12.7 (17)	64.7 (11)	35.3 (6)	4.21	2.42–7.30	
**Tuberculosis**						
**No**	97.8 (131)	19.8 (26)	80.2 (105)	1		**0.001**
**Yes**	2.2 (3)	100.0 (3)	0.0 (0)	5.04	3.57–7.11	
**Treatment**						
**Pentavalent Antimonial**	4.5 (6)	0.0 (0)	100.0 (6)	0.00	-	1.000
**Amphotericin B deoxycholate**	15.7 (21)	14.3 (3)	85.7 (18)	1.31	0.35–4.99	0.698
**Liposomal Amphotericin B**	45.5 (61)	34.4 (21)	65.6 (40)	3.17	1.29–7.77	**0.010**
**Association of two drugs**	34.3 (46)	10.9 (5)	89.1 (41)	1		

The results are presented as relative frequency (absolute frequency). RR = relative risk. CI 95% = Confidence interval of 95%. P values in the chi-square test. Significant risks are indicated by *p* values in bold.

**Table 2:** Adult patients with visceral leishmaniasis according to the results of laboratory tests at admission stratified by death (n = 134).

Variable	Death		*p* value
	Yes	No	
	Mean ± SD	Mean ± SD	
Erythrocytes (million/mm^3^)	3.07±0.15	3.48±0.08	**0.014**
Leukocytes (mil/mm^3^)	6,64±2,10	2,64±0,21	0.068
Rod cell (%)	12.31±1.56	15.51±1.22	0.110
Segmented neutrophils (%)	54.24±4.01	39.85±1.50	**0.002**
Eosinophils (%)	2.00±0.87	1.07±0.26	0.311
Basophils (%)	0.03±0.03	0.18±0.05	**0.023**
Lymphocytes (%)	23.72±3.45	32.41±1.43	**0.009**
Monocytes (%)	6.07±0.71	7.79±0.45	0.067
Platelets (million/mm^3^)	111,17±15,81	97,90±6,90	0.393
Albumin (g/dl)	2.67±0.20	2.98±0.07	0.060
Creatinine (g/dl)	1.28±0.17	1.02±0.07	0.165
Urea (mg/dl)	56.49±8.60	30.59±2.35	**0.007**
ALT (U/L)	75.55±35.12	53.19±5.30	0.534
AST (U/L)	88.03±14.91	67.75±7.52	0.216
Globulin	2.67±0.20	5.68±2.70	0.563
Total bilirubin	3.64±0.31	4.24±0.19	0.139
Na (mEq/ml)	137.00±1.22	135.69±0.50	0.259
K (mmol/L)	3.82±0.14	4.78±0.58	0.379
Glucose (mg/dl)	115.75±9.76	105.77±5.26	0.371

The results are presented as the mean ± standard error (SD). P values in the students t-test. The significant differences between patients who died and those that survived are shown with P values in bold.

**Fig 1:**
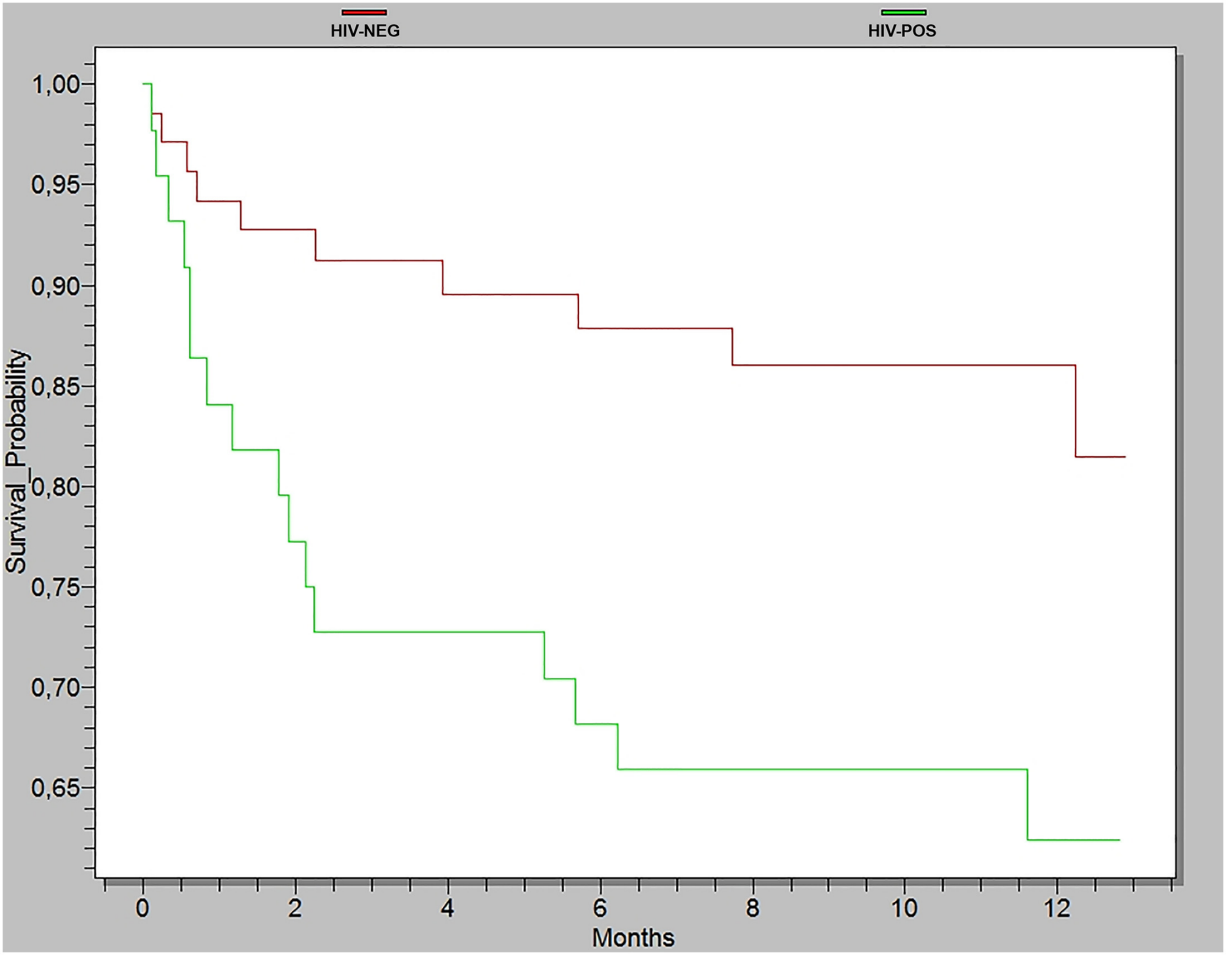
Adult survival, positive for visceral leishmaniasis, stratified by the presence (n = 49) or absence (n = 105) of HIV/AIDS infection during a 12-month follow-up period. Log-rank test = 11, 2534; p = 0,0008.

Patients with VL and HIV/AIDS exhibited a mortality rate of 36.7% (18/49) (Fig 1). Variables identified as risk factors for mortality were HIV/AIDS, relapse, secondary bacterial infection and edema (Table 3). Although splenomegaly presented a *p*<0.05 in the univariate analysis, it showed a strong correlation with HIV and when included in the final model and a reduction in the Wald test value when included in the multivariable model. Therefore, we only HIV variable was evaluated in the final model.

**Table 3:** Univariate and multivariate analysis and progress to death in adult patients with visceral leishmaniasis (n = 134).

	Univariate analysis			Multivariate analysis		
Variable	RR	95% CI	*p value*	RR	95% CI	*p value*
Secondary bacterial infection	7,10	3,11–16,20	<0,001	2.57	1.31–3.83	**<0,001**
Relapse	3,41	1,89–6,13	<0,001	5.87	1.39–24.75	**0.016**
Edema	3,29	1,64–6,57	<0,001	5.20	1.55–17.49	**0.008**
HIV/AIDS	3,30	1,67–6,51	<0,001	4.63	1,35–15.90	**0.015**

RR = relative risk. CI 95% = Confidence interval of 95%.

Multivariate analysis was followed by the Wald test and correlation matrix.

Among 23 patients who relapsed, 15 (65.2%) were HIV-positive. Although there was an association between HIV and relapse (*p*<0.01), the two variables in the final model had a higher Wald test value than when only one of the variables was included in the model. Of the 8 patients without HIV infection who relapsed, one had systemic erythematous lupus, one had leukemia, one had cirrhosis, and two were older than 85 years of age.

Of the 49 patients with AIDS, 7(14.3%) died during hospitalization and only 15/42 (35.7%) of them regularly adhered to secondary prophylaxis. No difference in relapse and death rates was observed among of those who adhered and those did not adhere to prophylaxis [33.3% versus 29.6%; *p* = 0.92 and 40.0% versus 22.2%; *p* = 0.38, respectively].

## Discussion

Most studies that evaluated risk factors for death among VL patients are retrospective design, secondary data analysis and involving different age groups [4, 6, 7, 8]. Our study differs by providing a 12-month follow-up of a cohort of adult patients from an urban center with a high co-infection rate of HIV/AIDS. In this way, relapses and deaths that occurred after the first episode of VL in this particular group of patients could also be documented.

The mortality rate (21.6%) observed in the present study is higher than the rates normally detected in Brazil [2, 3, 5]. This high rate could be associated with the clinical differences of patients included in the study who were mostly over 50 years of age and were co-infected with HIV/AIDS [4, 16, 17]. Herein, we identified four variables associated with death among VL patients: secondary bacterial infection, edema, HIV/AIDS co-infection and VL relapse.

Secondary bacterial infection is a well-known risk factor related to the severity of sepsis and described in previous studies [7, 8, 18, 19, 20]. The presence of edema, which may reflect protein malnutrition, liver or renal failure, has also been described as a risk factor for an unfavorable outcome [7, 8].

Recent studies have also identified that co-infection with HIV/AIDS is a risk factor for death [4, 5, 20]. The 36% mortality rate among HIV/AIDS patients, in this study, is much higher than studies previously conducted in Brazil [21, 22, 23, 24]. It is known that patients who are HIV/AIDS-positive are more likely to develop VL due to the depletion of both cellular and humoral immune responses against *Leishmania* [25] and, moreover, VL/HIV co-infection can accelerate the evolution of both diseases [26], given that the two agents are located in the same host cell. The co-infection can also enhance pathogenic effects and impair the correct function of macrophages [27, 28]. This synergism favors a fatal outcome of patients with VL.

Liposomal amphotericin B is the first choice of treatment in Brazil for the following individuals: VL positive patients over 50 years of age, patients co-infected with HIV/AIDS, patients with immunosuppressive diseases, and those with renal or cardiac disorders [13, 29]. Therefore, patients who used this drug were those affected with greater severity and a higher propensity towards death.

In the present study, the follow-up period was 12 months, which enabled the detection of relapses, deaths during relapse episodes and a correlation between HIV and relapses of VL. In general, co-infection with HIV/AIDS has a strong association with VL relapses [30] and the frequency of relapses in patients co-infected with VL-HIV/AIDS ranges from 10 to 60% [31]. A systematic review identified that the predictors of VL relapses in HIV-infected patients as: the absence of an increase in CD4+ T cells at follow-up, a lack of secondary prophylaxis, previous history of VL relapses, and CD4+ T cell counts below 100 cells/μl at the time of primary VL diagnosis [32].

Altered immunity may persist in co-infected patients, and the lymphocyte T-CD4+ count can be low, despite the administration of HAART (Highly Active Antiretroviral Therapy) and anti-leishmania therapy [33]. The cell activation increases the transcription of the integrated HIV that results in CD4+ cell death. It is induced by the activation of CD4+ and CD8+T cell memory population, resulting in an exhaustion of immune resources. After the treatment some parasites remains inside macrophages and leishmania antigens was thought to be responsible for the cellular activation observed in AIDS patients [27]. This additional monocyte/macrophage activation in VL/AIDS patients has been also associated with increased microbial translocation [34, 35].

According to the recommendations of the WHO, patients with HIV/AIDS should receive secondary prophylaxis to prevent a relapse of VL [32, 36, 37]. Although secondary prophylaxis was indicated, in our study, for all patients with a T-CD4+ count lower than 350 cell/mm^3^, adherence to secondary prophylaxis was not associated with protection of relapse or death during the follow-up. Other studies have shown that secondary prophylaxis is not completely effective in prevent relapses [38, 39].

It is possible that the VL treatment used in patients with HIV infection has been insufficient to reduced parasitic load and cure. During the study period, the recommended liposomal amphotericin B total dose was 20mg/kg. However the current recommendation by the Ministry of Health of Brazil is 40mg/kg, instead of 20mg/kg, in patients co-infected with HIV [15, 40]. Although the cure criteria are essentially clinical, many clinical and laboratory changes are still present, including splenomegaly, anemia and hypergammaglobulinemia, at the end of treatment in co-infections patients. There is no recommendation to repeat the parasitological examination at the end of treatment [41] and Real-time PCR has been proposed as a suitable tool for monitoring the parasite load during follow-up of co-infected patients and predict the risk of relapses after treatment [42].

In conclusion, this study demonstrated that VL is a serious disease with high mortality rates in adult patients from urban areas, especially when co-infected with HIV/AIDS. Further studies are needed to define the best therapeutic options for effective treatment and prevent VL relapses in these patients.

## Supporting Information

S1 Data Dataset containing variables of the study. (XLS)

S1 Checklist STROBE Checklist. (DOCX)
